# An air-liquid interphase approach for modeling the early embryo-maternal contact zone

**DOI:** 10.1038/srep42298

**Published:** 2017-02-09

**Authors:** S. Chen, S. E. Palma-Vera, M. Langhammer, S. P. Galuska, B. C. Braun, E. Krause, A. Lucas-Hahn, J. Schoen

**Affiliations:** 1 Leibniz Institute for Farm Animal Biology, FBN Dummerstorf, Germany; 2 Institute of Veterinary Biochemistry, Freie Universitaet Berlin, Germany; 3 Leibniz Institute for Zoo and Wildlife Research, IZW Berlin, Germany; 4 Leibniz Institute for Molecular Pharmacology, FMP Berlin, Germany; 5 Institute of Farm Animal Genetics, Federal Research Institute for Animal Health, Mariensee, Germany

## Abstract

We developed an air-liquid interphase culture procedure for mammalian oviduct epithelial cells leading to the formation of functional epithelial tissues, which generate oviduct fluid surrogates. These *in vitro* oviduct epithelia can be co-cultured with living zygotes and enable embryonic development up to the blastocyst stage without addition of embryo culture medium. The described strategy is broadly applicable to analyze early embryo-maternal interactions under standardized *in vitro* conditions.

Embryo-maternal interactions are necessary to initiate and maintain pregnancy and are supposed to be causal for early embryonic mortality (the most common reproductive failure in mammals) upon their disturbance^1^. Investigating the subtle local interactions between the mammalian pre-implantation embryo and the maternal organism is technically challenging if not impossible *in vivo*. The first maternal “contact zone” for the early embryo is the oviduct epithelium, a simple, columnar shaped and ciliated epithelium, which is structurally and functionally defined by polarized distribution of organelles and proteins. Function, growth and survival of epithelial cells correlate with their degree of polarity^2^,^3^. However, when grown under standard culture conditions (adherent submerged or suspension culture) oviduct epithelial cells (OEC) do not maintain an epithelial phenotype for prolonged culture periods and exhibit marked changes in their morphological and functional integrity^4^,^5^. To solve this problem we applied an air-liquid interphase (ALI) system to culture porcine OEC resulting in the formation of highly differentiated *in vitro* models^6^,^7^. While applications for human and later for bovine ALI-OEC have been established by other groups^8^,^9^, so far, embryo development could not be demonstrated in co-culture on these model systems.

For this purpose, we developed the first ALI culture strategy appropriate for long-term co-culture of ALI-OEC with developing embryos, requiring no embryo culture medium. We validated applicability of the procedure for murine, porcine and bovine OEC (MOEC, BOEC and POEC, respectively), since these species are frequently used model systems in reproductive biology.

The ALI culture procedure consisted of a proliferation phase and a differentiation phase (Fig. 1). Murine oviducts were enzymatically digested following a protocol reported for murine tracheal cells^10^. POEC and BOEC were isolated as described previously^6^,^11^. Isolated OEC were seeded on culture inserts and first grown under submerged conditions in a proliferation-inducing medium for 7 d. After the proliferation phase (confluency reached) the medium was suctioned off the apical compartment. Cells were maintained under serum free (MOEC and POEC) or serum-reduced (3% FBS, BOEC) conditions with provision of growth medium from the basolateral compartment only and allowed to differentiate morphologically and functionally for another two (POEC and MOEC) or three weeks (BOEC). During the differentiation period ALI-OEC developed from flat cell layers to columnar shaped epithelia consisting of secretory and ciliated cells (Fig. 2) and maintained this differentiation status up to week eight, when the experiment was terminated (Fig. 3A–C). The epithelial layer build up a barrier function (as measured by trans-epithelial electrical resistance, TEER, Fig. 3D), which reached constant values after three (ALI-MOEC and -POEC) or four (ALI-BOEC) weeks of culture and stayed stable up to week eight.

After the differentiation period ALI-MOEC, -BOEC and -POEC formed an oviduct fluid surrogate (OFS) in the apical insert compartment (20–30 μl in ALI-MOEC, 10–20 μl in ALI-POEC and -BOEC in a 24 well insert format).

To prove reproducibility of OFS formation, we tested its osmolality, its protein pattern in SDS-PAGE, and secretion of the marker protein oviductin (OVGP1) in five biological replicates per species. The OFS of all three species exhibited an *in vivo*-like osmolality (ALI-MOEC: 341 ± 4.4 mOs/kg, ALI-POEC: 348 ± 8.3 mOs/kg, ALI-BOEC: 340 ± 3.5 mOs/kg) with low variability. SDS-PAGE showed a reproducible species specific protein pattern (Fig. 4A). Oviductin is a marker-mucin almost exclusively expressed in oviduct epithelium. It is involved in zona pellucida hardening as well as sperm-oocyte interaction. Under standard *in vitro* conditions its expression was rapidly down-regulated^4^,^5^,^12^. Under ALI conditions, however, it was abundant in OFS from all three species and secreted in different glycosylated (and therefore potentially functional) forms as shown by N-glycosidase F (PNGaseF; cleaves complete N-glycans) and sialidase (releases sialic acids) treatment (Fig. 4B,C).

To characterize the protein content of the OFS we performed proteome analysis by mass spectrometry. Murine, bovine and porcine OFS contained proteins previously reported to be highly abundant in oviductal fluid *in vivo*^13^ (table of most abundant OFS proteins: Suppl. Table 1; complete proteome data: Suppl. Table 2). Along with OVGP1 many other proteins, which are proven to influence fertilization and/or embryonic development (e.g. osteopontin, components of the plasminogen/plasmin system, heat shock proteins, lactoferrin) are present in the oviductal fluid surrogate (OFS) formed by the ALI-OEC systems. In total 1756, 2979 and 3094 proteins were identified in murine, porcine and bovine OFS, respectively. The only fully available, comprehensive proteome study of *in vivo* oviductal fluid was conducted in sheep and was very recently published^13^. Taking this data set as reference ~97% (murine: 96.9%; porcine: 96.3%; bovine: 96.8%) of the proteins detected in the OFS are also abundant in the sheep oviductal fluid *in vivo*.

To finally prove the functional integrity of the cell culture system, we tested the capability of the *in vitro* formed epithelial tissue to support embryo development in long-term co-culture. Zygotes were either produced *in vitro* (IVM/IVF; bovine) or *in vivo* (mouse and pig). After IVF or flushing from the *in vivo* oviducts, potential zygotes were briefly washed in PBS and placed in the OFS on top of the ALI-OEC in groups of 10–30 (Suppl. Video 1). Two experimental setups were conducted in each species. First, co-cultures were terminated on d 2 to determine cleavage rate. Second, we prolonged embryo co-culture to test whether the milieu of the OFS provided by the ALI-OEC supports further embryonic development (mouse: 4.5 d, pig 7 d and cattle 8 d). In all three species cleavage as well as blastocyst formation could be observed without supplementation of any embryo culture medium (Table 1, Fig. 5).

In sum, we developed a culture procedure for the formation of an *in vivo*-like oviduct tissue substitute from primary oviduct epithelial cells. We demonstrated that the formed tissue is fully functional in terms of morphological differentiation (polarization, columnar shape, ciliary activity) and in terms of oviductal fluid surrogate formation supporting embryo development *in vitro* without additional embryo culture medium supply.

The blastocyst rates in co-culture could not yet match the outcome of optimized standard IVEP procedures. Therefore the model could be further improved by a) simulation of the hormonal changes taking place during the periconceptional period and b) development of a sequential culture system using oviductal as well as uterine epithelial cells. This might increase the efficiency of the system both quantitatively and qualitatively. Further experiments including *in vivo* embryo transfer then have to be conducted to assess the quality of ALI-produced blastocysts.

The presented culture strategy is broadly applicable to analyze early embryo-maternal interactions under standardized *in vitro* conditions. It can prospectively serve as a tool to advance IVEP procedures by analyzing specific components of the dynamic oviduct milieu regarding their impact on the early embryo. This might facilitate the development of new strategies (e.g. media supplements) for livestock as well as for human ARTs.

## Methods

### Media and reagents

DMEM/Ham’s F12, FBS, HEPES, penicillin/streptomycin and amphotericin B were purchased from Merck Millipore, while other reagents were obtained from Sigma unless otherwise indicated. Media used in this culture procedure are modifications of a protocol reported for mouse tracheal cells [10]. The basic medium consisted of DMEM/Ham’s F12, 15 mM HEPES, 100 U/ml penicillin, 100 μg/ml streptomycin and 0.25 μg/ml amphotericin B. The proliferative medium (M1) for d 0–d 7 was basic medium supplemented with 5% FBS, 10 μg/ml insulin, 5 μg/ml transferrin, 0.1 μg/ml cholera toxin, 25 ng/ml epidermal growth factor, 30 μg/ml bovine pituitary extract. Differentiation medium (M2, from d 7 onwards) for ALI-MOEC and -POEC (M2a) was serum free: basic medium supplemented with 1 mg/ml BSA, 5 μg/ml insulin, 5 μg/ml transferrin, 0.025 μg/ml cholera toxin, 5 ng/ml epidermal growth factor, 30 μg/ml bovine pituitary extract. For ALI-BOEC the differentiation medium (M2b) consisted of basic medium with 3% FBS and 2% Nuserum (Corning). All culture media were freshly added with 0.05 μM retinoic acid directly before use.

### Animals

The FBN mice strain Fzt:DU^14^ was included in this study. Porcine and bovine tissue samples were slaughterhouse by-products and collected in local abattoirs. All animal procedures were done in accordance to national and international guidelines and approved by the institutional Animal Protection Board at FBN.

### Isolation and culture of MOEC

For the isolation of MOEC 6–8 weeks old female mice were killed in oestrus (detected by vaginal cytology). Both oviductal tubes were resected from the reproductive tract, washed in cold basic medium, combined and sliced into small pieces and incubated in basic medium with 0.15% Pronase at 4 °C overnight. The next day the sample was centrifuged at 250 × g for 8 min and the pellet was subsequently digested in 300 μl of 0.5 mg/ml DNase I for 10 min. After passing through a 100 μm cell strainer, the dissociated cells were centrifuged and re-suspended in M1 medium. The cell yield from each mouse was approximately 1–3 × 10^5^ epithelial cells.

**Figure 1:**
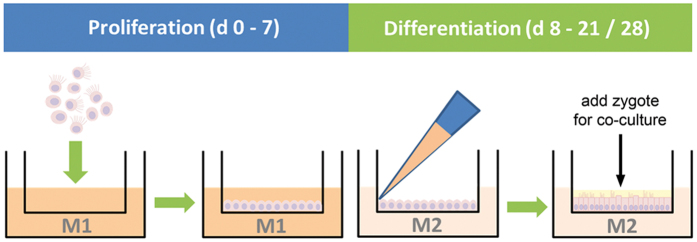
Scheme of the two-step ALI-OEC establishment procedure.

A schematic diagram for the insert-supported culture is shown in Fig. 1A. 24-well inserts with a pore size of 1.0 μm (Merck Millipore) were coated with 100 μl/insert human placental collagen (1 mg/ml) overnight and washed three times with PBS afterwards. 1–1.5 × 10^5^ cells isolated from single mice were seeded per insert. From d 0 to d 6 (proliferation phase), cells were held submerged in medium with 200 μl of M1 medium in the apical compartment and 1 ml of M1 in the basal compartment. From d 7 onwards (differentiation phase) cells were switched to air-liquid interphase (ALI), with access to M2a medium only in the basal compartment. Cultures were maintained at 37 °C, 5% CO_2_, with medium refreshment twice per week. Apical fluid was removed from the upper compartment during each medium change.

### Isolation and culture of POEC and BOEC

Isolation of oviductal cells from large farm animals as pig and cattle was performed as previously reported by our group^6^,^11^. Briefly, oviducts were collected from a local slaughterhouse and transported immediately on ice for laboratory processing. After washing in PBS, each oviductal tube was filled up with 1 mg/ml collagenase 1 A and incubated for 1 h at 37 °C. Big epithelial clusters were collected with a cell strainer and then further digested in accutase (Life technologies) for 10 min. Thereafter cells were centrifuged and re-suspended in M1 medium for seeding.

While the culture of POEC was performed as described for MOEC (see above), for BOEC the procedure had to be modified: 1. Cells were maintained on 0.4 μm-pore-size 24 well inserts (no collagen coating); 2. Medium for the differentiation phase was M2b.

### Histology and immunofluorescence

The preparation of tissue and inserts for histology has been described previously^15^. After embedding, 4 μm paraffin sections were cut for haematoxylin/eosin (HE) and immunofluorescence staining (N = 5 from different donor animals per species). Antigen retrieval was carried out by cooking slides in 10 mM citrate buffer. The primary antibodies were rabbit anti-beta-Catenin (1:1000, Cell Signalling 9562) and mouse anti-acetylated tubulin (1:1000, Sigma T7451). Goat anti-mouse Alexa 488 (1:40, Invitrogen A11017) and goat anti-rabbit IgG Alexa 546 (1:200, Invitrogen A11071) were used as secondary antibodies. Nuclei were counterstained with TO-PRO-3 (1:500, Invitrogen T3605). Images were captured by the confocal laser scanning microscope LSM 800 equipped with ZEN software (Carl Zeiss).

### Transepithelial electrical resistance assessment

Before harvesting the ALI cultures, transepithelial electrical resistance (TEER) was measured by the EVOM2 Epithelial Voltohmmeter (WPI) following the manufacturer’s instructions (N = 5 from different donor animals per species).

### Characterization of oviduct fluid surrogates (OFS)

#### SDS PAGE, Western blot, immunodetection of oviductin (OVGP1) and oviductin glycosylation analysis

On d 21 (ALI-MOEC and -POEC) or d 28 (ALI-BOEC) of culture, apical fluid was suctioned off. 3–5d later OFS was collected for analysis. The collected fluid was centrifuged at 250 × g for 8 min to remove cell fragments and the supernatant was immediately kept at −70 °C. 10–30 μl of OFS could be recovered from each 24-well insert. OFS from ALI-MOEC, -POEC and -BOEC (5 different donor animals per species) were run on SDS-PAGE gels (Commassie staining: 10 μl OFS, 12% gel; Western Blot: 5 μl OFS, 7.5% gel). Oviductin was detected after Western Blot (1:1000, Santa Cruz sc-46430) and visualized by a POD-coupled secondary antibody (1:3000, Santa Cruz sc-2020) and chemiluminescence (ECLprime, GE Healthcare). To test for oviductin glycosylation OFS samples were incubated (37 °C, overnight) with sialidase (Neuraminidase from *vibrio cholerae*, 0.15 mU/μl, Roche) or PNGaseF (70 U/μl, New England BioLabs), respectively, before SDS-PAGE, Western Blot and oviductin detection.

#### Protein identification by LC-MS/MS

The OFS recovered from ALI-MOEC, -POEC and -BOEC (N = 1 per species) were run on a SDS-PAGE gel and cut into 13 equal-sized Coomassie-stained bands. In-gel protein digestion was performed as previously described^16^. Digested samples were re-dissolved in 0.1% TFA and 5% acetonitrile, and peptides were analyzed by a reversed-phase capillary liquid chromatography system (Ultimate 3000 nanoLC system, Thermo Scientific) connected to an Orbitrap Fusion mass spectrometer (Thermo Scientific). LC separation was performed on an in-house packed 75 μm inner diameter PicoTip column (25 cm) packed with ReproSil-Pur C18AQ particles, 3 μm, 120 Å (Dr. Maisch). The flow rate was 200 nL/min using a gradient of 3−30% B in 60 min. Mobile phase solvent A contained 0.1% formic acid in water and mobile-phase solvent B contained 0.1% formic acid in acetonitrile. For MS/MS measurements, FT survey scans were acquired with a resolution of 120.000. The data-dependent acquisition (DDA) mode and monoisotopic precursor ions with charge states 2 and 3 were selected. HCD MS/MS spectra were acquired in the linear ion trap using a quadrupole isolation window of 1.6 Da.

Protein identification was performed using Mascot Distiller (version 2.5.1.0). Processed data were searched against a SwissProt database (version 2014_12; 547,085 sequences). The mass tolerance of precursor and sequence ions was set to 10 ppm and 0.35 Da, respectively. A maximum of two missed cleavages was allowed. Methionine oxidation and the acrylamide modification of cysteine were used as variable modifications. Scaffold (version 2.01; Proteome Software Inc.) was used to validate MS/MS based peptide and protein identifications. Peptide identifications were accepted if their probability was established at > 70%, as specified by the Peptide Prophet algorithm. Protein identifications were accepted if their probability was established at > 90% and if they contained at least two identified tryptic peptides.

#### Osmolality of OFS

The osmolality of apical fluids collected from ALI-MOEC, -POEC and -BOEC, was measured using the cryoscopic osmometer (Gonotec) according to the manufacturer´s instructions. Measurement was performed with 15 μl of apical fluid (N = 5 inserts, 3–5 different donor animals per species).

### Embryo co-culture

#### ALI-OEC preparation before Co-culture

On culture day d 21 (ALI-MOEC and -POEC) or d 28 (ALI-BOEC) apical fluid was suctioned off the insert, 3d (ALI-MOEC) or 5d (ALI-POEC and -BOEC) later around 10–30 μl fresh apical fluid was regenerated inside the insert. As co-culture was carried out at 37 °C (murine zygotes) or 38.5 °C (porcine and bovine zygotes) in a humidified atmosphere of 5% O_2_ and 5% CO_2_, ALI-POEC and -BOEC were step-wise adapted to these conditions over 2–3 days before co-culture.

#### Zygote production

##### Mice

20 virgin females of the mouse line Fzt:DU (10–12 weeks old) were randomly selected. Potential zygotes were collected from the oviducts of naturally mated female mice approx. 12 h post conception (vaginal plug).

##### Pig

For collection of *in-vivo* derived porcine embryos, in total 10 sows were slaughtered and potential zygotes and 2-cell embryos were recovered approximately 12 h after artificial insemination.

##### Cattle

Bovine ovaries were collected from a local slaughterhouse and transported in DPBS with 1% penicillin to the laboratory within 3 h after slaughter. Upon arrival, cumulus oocyte complexes (COCs) were recovered using DPBS supplemented with 0.2% BSA. The COCs were matured in Medium 199 supplemented with 5% estrous cow serum, 0.07 IE/ml FSH, 0.03 IE/ml HCG, 1 μg/ml estradiol, 2 mM L-glutamine and 1% penicillin/streptomycin at 38.5 °C (5% CO_2_) for 24 h. After maturation, 10 COCs were transferred to each 100 μl droplet of fertilization medium. Motile sperm were recovered by swim-up separation of frozen-thawed semen. 3 × 10^4^ sperm were added to each fertilization droplet (Tyrode’s albumin lactate pyruvate medium) and incubated with the matured COCs for 18 h.

#### Co-Culture

On d 24 (ALI-MOEC), d 26 (ALI-POEC) or d 33 (ALI-BOEC) of culture potential zygotes of all three species were transferred to the apical side of the respective OEC cultures in groups of 10–30.

As exact evaluation of cleavage is difficult on the inserts, three experimental trials were performed: in trial 1 and 2 the experiment was terminated on d 2 of co-culture to determine the percentage of cleaved embryos. In trial 3 embryos were further cultured until d 4.5 (mice), 7 (pig) or 8 (cattle) to evaluate if embryonic development was further supported by ALI-OEC. Blastocysts were fixed in buffered formol saline (4%) and stained with Hoechst 33258.

## Additional Information

**How to cite this article**: Chen, S. *et al*. An air-liquid interphase approach for modeling the early embryo-maternal contact zone. *Sci. Rep.* **7**, 42298; doi: 10.1038/srep42298 (2017).

**Publisher's note:** Springer Nature remains neutral with regard to jurisdictional claims in published maps and institutional affiliations.

## Supplementary Material

Supplementary Information

Supplementary Table 1

Supplementary Table 2

Supplementary Video 1

## Figures and Tables

**Figure 2:**
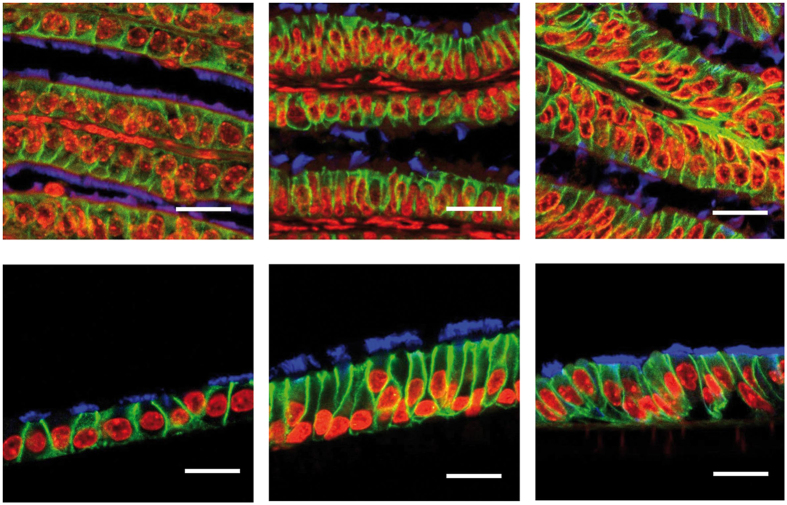
Oviduct epithelial cells of different mammalian species grown at the air-liquid interphase (ALI). Immunodetection of epithelial markers in murine, porcine and bovine oviduct tissue (from left to right; upper pictures) and respective ALI-OEC (lower pictures) after 21 d (ALI-MOEC and -POEC) or 28 d (ALI-BOEC) of culture. Red: nuclei; green: beta-catenin (cell-cell adhesions); blue: acetylated tubulin (cilia); bar = 10 μm.

**Figure 3:**
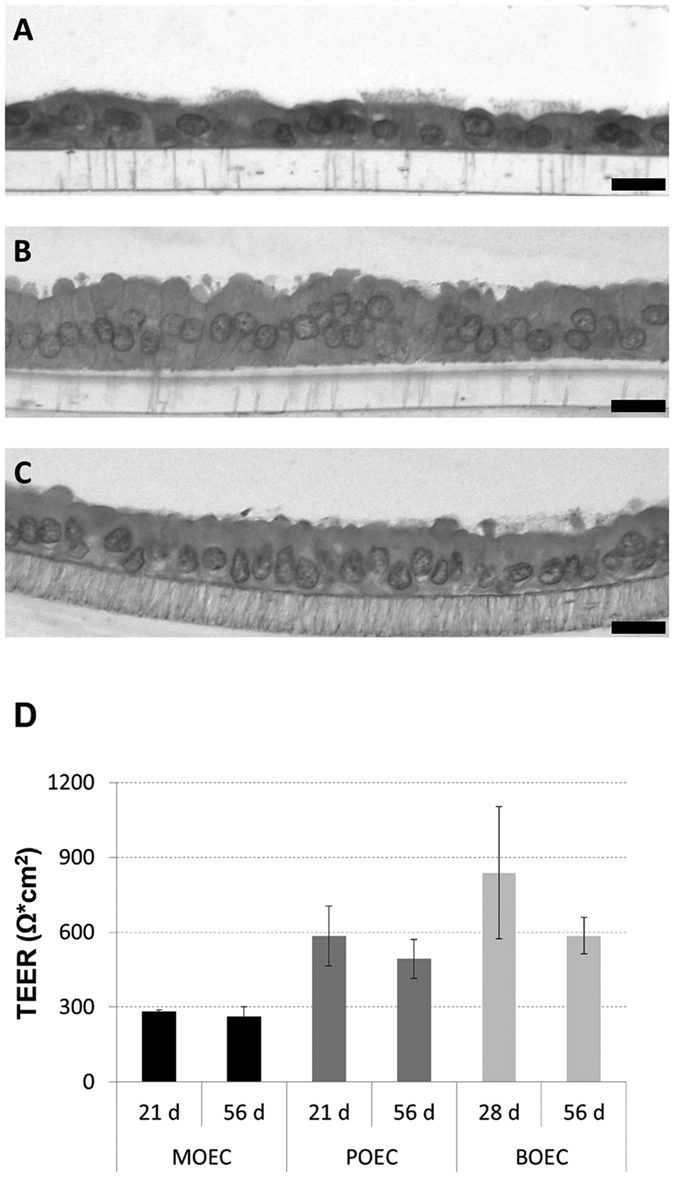
Characterization of ALI-OEC in long-term culture. (**A–C**) Murine, porcine and bovine ALI-OEC (from top to bottom) after 56 d in culture. HE staining; magnification x400. Bar = 10 mm. (**D**) TEER of murine, porcine and bovine OEC during long-term culture at the ALI.

**Figure 4:**
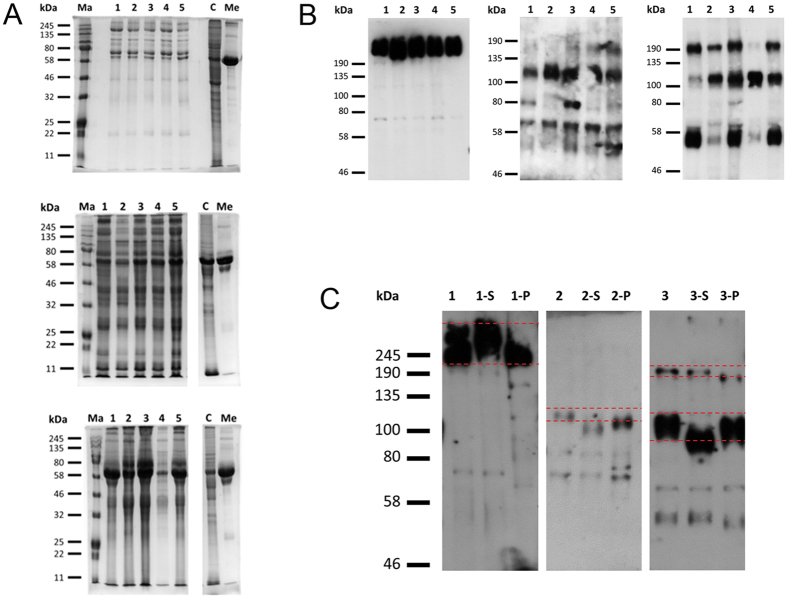
Protein characterization in oviductal fluid surrogates (OFS). (**A**) Protein patterns in murine, porcine and bovine OFS (from top to bottom). Five biological replicates. Ma = marker; 1–5 = animal 1–5; C = cell lysate (epithelial cells after isolation from oviduct tissue); Me = growth medium. (**B**) Immunodetection of oviductin (OVGP1) in murine, porcine and bovine (from left to right) OFS after SDS-PAGE and Western Blot. 1–5 = OFS from donor animal 1–5. (**C**) Immunodetection of oviductin (OVGP1) in murine, porcine and bovine OFS after glycosidase digestion, SDS-PAGE and Western Blot. 1 = murine, 2 = porcine, 3 = bovine OFS, respectively; -S = digested with sialidase; -P = digested with PGNaseF.

**Figure 5:**
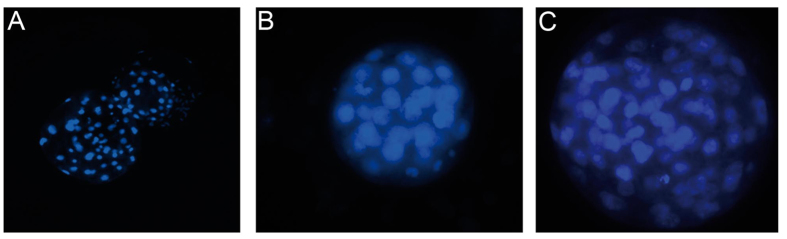
Mammalian embryos produced by co-culture with ALI-OEC. (**A**) Hatching porcine blastocyst (d 7; Hoechst staining; magnification x100). (**B**) murine blastocyst (d 4.5; Hoechst staining; magnification x400). (**C**) Bovine blastocyst (d 8; Hoechst staining; magnification x400).

**Table 1:** Zygote development on murine, porcine and bovine ALI-OEC.

	Trial	No. of zygotes*	Cleavage rate % (No. of ≥2cell embryos)**	Blastocyst rate% (No. blastocyst)**
**MOEC**	**1**	49U	67.35 (33)	—
	**2**	13U	92.31 (12)	—
	**3**	19U	—	52.63 (10)
**POEC**	**1**	21U + 20II	90.24 (37)	—
	**2**	35U + 3II	84.21 (38)	—
	**3**	57U + 35II	—	48.91 (45)
**BOEC**	**1**	109U	66.10 (72)	—
	**2**	121U	56.20 (68)	—
	**3**	98U	—	7.14 (7)

^*^U = uncleaved potential zygotes, II = 2-cell embryos.

^**^Cleavage was assessed after 2 days, and blastocyst rate after 4.5 (mouse), 7 (pig) or 8 (cattle) days of co-culture on ALI-OEC.
