# Supplementary material for: An air-liquid interphase approach for modeling the early embryo-maternal contact zone
Source: Sci Rep. 2017 Feb 9;7:42298. doi: 10.1038/srep42298 (PMC5299422; doi:10.1038/srep42298)
Supplement: Supplementary Information [file srep42298-s1.pdf]

Supplementary Information

An air-liquid interphase approach for modeling the early embryo-maternal contact zone

Chen S[1], Palma-Vera SE[1,2], Langhammer M[1], Galuska SP[1], Braun BC[3], Krause E[4], Lucas-Hahn A[5] and Schoen J[1*]

*Corresponding author (schoen.jennifer@fbn-dummerstorf.de)

Supplementary legends:

Suppl. Table 1: Most abundant proteins in murine, porcine and bovine oviductal fluid surrogates.

Suppl. Table 2: Peptides identified in murine, porcine and bovine oviductal fluid surrogates.

Suppl. Video 1: Developing porcine embryo on ALI-POEC.
